# Diagnosis of Murine Typhus by Serology in Peninsular Malaysia: A Case Report Where Rickettsial Illnesses, Leptospirosis and Dengue Co-Circulate

**DOI:** 10.3390/tropicalmed4010023

**Published:** 2019-01-31

**Authors:** Yazli Yuhana, Ampai Tanganuchitcharnchai, Pimpan Sujariyakul, Piengchan Sonthayanon, Kesinee Chotivanich, Daniel H. Paris, Sasithon Pukrittayakamee, Stuart D. Blacksell, Borimas Hanboonkunupakarn

**Affiliations:** 1 Department of Clinical Tropical Medicine, Faculty of Tropical Medicine, Mahidol University, Bangkok 10400, Thailand; aleeyuhana@hotmail.com (Y.Y.); nok@tropmedres.ac (K.C.); sasithon@tropmedres.ac (S.P.); 2 Infectious Diseases Unit, Department of Internal Medicine, Universiti Teknologi MARA, Sungai Buloh 47000, Malaysia; 3 Mahidol-Oxford Tropical Medicine Research Unit, Faculty of Tropical Medicine, Mahidol University, Bangkok 10400, Thailand; Ampai@tropmedres.ac (A.T.); Pimpan@tropmedres.ac (P.S.); 4 Department of Molecular Tropical Medicine and Genetics, Faculty of Tropical Medicine, Mahidol University, Bangkok 10400, Thailand; piengchan@tropmedres.ac; 5 Department of Medicine, Swiss Tropical and Public Health Institute, 4056 Basel, Switzerland; daniel.paris@unibas.ch; 6 Faculty of Medicine, University of Basel, 4003 Basel, Switzerland; 7 Centre for Tropical Medicine, Nuffield Department of Clinical Medicine, Churchill Hospital, Oxford OX3 7FZ, UK

**Keywords:** murine typhus, diagnosis, serology, Malaysia

## Abstract

Murine typhus is a rarely diagnosed cause of acute febrile illness in Malaysia, and its true disease burden is unknown. We report a case of an acute murine typhus infection in a patient living in a small city in Peninsular Malaysia, presenting with fever, rash, and headache. Unresponsive to the initial empirical treatment for leptospirosis, he showed a rapid response to doxycycline when murine typhus was diagnosed later. This case highlights the importance of considering murine typhus as a diagnostic in cases of acute febrile illness in urban and sub-urban areas, such as that of in Peninsular Malaysia.

## 1. Introduction

Murine typhus, caused by the obligately intracellular organism, *Rickettsia typhi*, is a disease transmitted via the rat flea, *Xenopsylla cheopis*, to humans. The disease has been historically long-known but remains under-appreciated and under-diagnosed, likely due to non-specific clinical presentation and the laborious nature of its diagnostic requirements [1,2]. The indirect immunoperoxidase (IIP) and immunofluorescence assays (IFA) are considered as the gold standard serological tests, the primary difference between the two being the visualization method of endpoint results via light (IIP) and fluorescence (IFA) microscopy, respectively [3,4]. 

Bites from rat fleas do not elicit eschars, unlike those from scrub typhus and spotted fever rickettsial typhus, which may assist in early clinical recognition [5,6]. However, similar to other rickettsioses, murine typhus may cause severe infections with multiorgan dysfunctions such as pneumonitis, meningitis, septic shock, and death [7,8,9,10,11,12]. Tetracycline group antibiotics such as doxycycline remain the mainstay of therapy, which are often readily available [13]. In Malaysia, murine typhus has demonstrated seroprevalence ranging between 2–30% in patients presenting to public hospitals [3]. 

We describe a patient whom was presented with undifferentiated symptoms, thrombocytopenia, and elevated hepatic transaminases who had initially been unsuccessfully treated for leptospirosis before a consideration of murine typhus was made.

## 2. Case Report 

During the rainy season in January 2016, a 39-year-old Indian man from Teluk Intan, Perak (Northwest of Peninsular Malaysia), complained of 10 days of fever, associated with headache, generalized myalgia, and rash over the lower limbs. There was no history of antibiotic therapy before the rash’s onset. He suffered no vomiting, visual complaints, confusion, or neck pain. The patient was a lorry driver, working for an oil-palm plantation and had frequent contact with rats around the oil-palm factories. There was no report of recent travel and involvement in forest or water-based recreational activities. His medical history was otherwise not significant. 

Upon physical examination, he was alert and orientated. His vital signs included an oral temperature of 39 °C, blood pressure of 110/77 mmHg with pulse rate of 88 beats per minute, respiratory rate of 18 breaths per minute and oxygen saturation of 98% on room air, with a score of 0 by using the quick sepsis related organ failure assessment (qSOFA) scoring [14]. Skin examination revealed erythematous macular papular rashes over both lower limbs, which spared to the palms and soles. There were no visible eschars. Eyes were normal and there were no palpable cervical lymph nodes, mouth ulcers, or myositis elicited. Cardio-respiratory examination was unremarkable, and abdominal examination failed to demonstrate organomegaly. 

The initial laboratory parameters demonstrated white cell counts of 7.9 × 10^9^ cell/L (reference range 4.0–12.0 × 10^9^ cell/L), mild thrombocytopenia of 126 × 10^9^ cell/L (reference range 150–440 × 10^9^ cell/L), normal serum creatinine level of 99 µmol/L (reference range 62–106 µmol/L), and hyponatremia at 127 mmol/L (reference range 136–145 mmol/L). Both liver enzymes were moderately elevated, with alanine aminotransferase of 131 IU/L (reference range 10–50 IU/L) and aspartate aminotransferase of 156 IU/L (reference range 10–40 IU/L). Normal total bilirubin level was 14.5 µmol/L (reference range ≤ 21 µmol/L) and mild hypoalbuminemia of 33 g/L (reference range 35–52 g/L). C-reactive protein (CRP) and procalcitonin level (PCT) were elevated at 80.23 mg/L (reference range < 5 mg/L) and 0.98 ng/mL (reference range < 0.5 ng/mL), respectively. 

**Figure 1:**
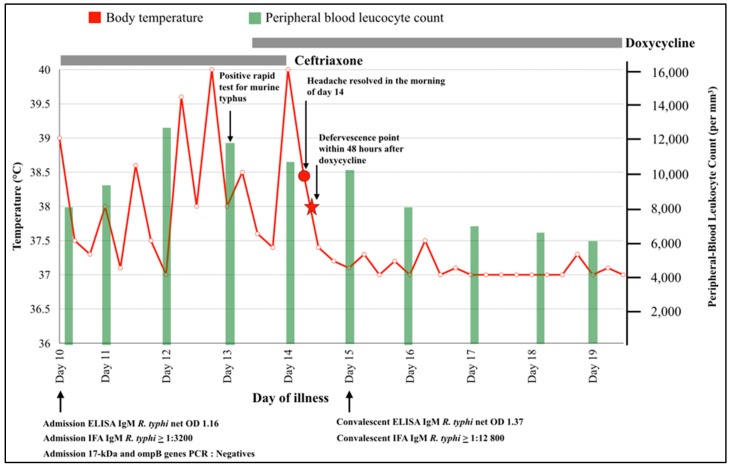
Clinical course of the 39-year-old patient with acute murine typhus infection.

In view of the clinical history and demographic considerations, the patient was treated empirically for leptospirosis using intravenous ceftriaxone, (2 g once a day). However, the initial leptospiral microagglutination test (MAT; using three local serovar and 17 serovar recommended by WHO) [15], dengue NS1, IgM and IgG (PanBio Dengue Capture ELISA), blood films for malaria and blood culture taken upon admission (BACTEC 9240, Bactec) were all negative. Furthermore, there was persistence in symptoms and worsening septic parameters, despite antibiotic therapy, which prompted the need to reconsider the initial diagnosis, one of which included murine typhus and other rickettsial diseases (Figure 1). Serological tests using rapid diagnostic kits for both scrub typhus (InBios scrub typhus IgM) and murine typhus (GenBio *R. typhi*) were both performed and yielded positive results in the later test. Ceftriaxone treatment was ended and oral doxycycline was initiated, with a loading dose of 200 mg followed by 100 mg twice a day for seven days (Figure 1). Symptom relief was achieved within 24 h of doxycycline administration. (Figure 1). 

Initial sera samples collected on the day of admission (day 10 of illness) were tested by IFA for IgM and IgG against Orientia tsutsugamushi (Karp, Kato, Gilliam and TA716 strains), R. typhi (Wilmington strain), and spotted fever group (R. felis strain) as described previously [16,17]. This revealed 1:3200 murine typhus IgM and 1:100 IgG in the IFA. Repeat convalescent sera sample collected five days later, revealed further increase of both IgM (1:12800) and IgG (1:800) (Figure 1). Comparatively, IFAs for scrub typhus IgM and IgG were <1:100 for both samples and spotted fever group rickettsia IFA resulted in 1:100 IgM and IgG <1:100, compared to 1:800 IgM and IgG <1:100 in the convalescent sample. Nucleic acid detection assays targeting the 47 kDa and 56 kDa outer membrane proteins of *O. tsutsugamushi* for scrub typhus and Lipl32, respectively, as well as the 16s rRNA for leptospirosis, and 17 kDa ompB genes of R. typhi were all negative. The patient completed seven days of doxycycline with blood parameter normalization and was subsequently discharged in a healthy condition (Figure 1).

### Ethics Statement

This study had been approved by the Malaysian National Medical Research and Ethics Committee (1 September 2015). An informed consent was signed by the patient.

## 3. Discussion

Following bites from infected rat fleas the *Xenopsylla cheopis*—the principal vector in murine typhus—patients often present with abrupt onset of fever (normally 8–16 days) following the inoculation of *R. typhi* [18,19]. Other common symptoms described include rashes and headache, similarly seen in cases of scrub typhus, leptospirosis, dengue, and malaria [19,20]. Studies in South-East Asia countries and their surrounding areas have demonstrated considerable prevalence of murine typhus as an important, treatable acute febrile illness especially within urban and suburban areas due to high density of the disease’s principal reservoirs; roof rats (*Rattus rattus*) and Norwegian rats (*Rattus norvegicus*) [7,10,19,20]. Diagnosis heavily relies on serological evidence via paired sera which should demonstrate at least a four-fold increase in specific antibodies [1]. Unfortunately, unlike scrub typhus there has been little advancements in rapid tests and ELISA testing for murine typhus [2,21,22]. Current commercially available rapid tests for murine typhus are both limited and poorly studied. One example, the GenBio rapid test kit is a semi-quantitative enzyme immunoassay targeting both IgG and IgM anti-bodies to *R. typhi* [23]. To our knowledge, this is the first reported case of acute murine typhus infection in Malaysia diagnosed by the rapid test kit, with subsequent confirmation via IFA of a high IgM titer in both admission (day 10 illness) and convalescent sample (1:3200 to 1:12800). IgM against *R. typhi* is invariably absent in the first week of illness, thus a paired serum sample is required to demonstrate a four-fold increase in IgM, indicating robust evidence of acute infection [16,24]. In regions where other rickettsial diseases co-circulate, a case of murine typhus co-infection with scrub typhus has been described by Laos [25]. In our case, we have ruled out co-infection with scrub typhus by using IFA and PCR against *O. tsutsugamushi* indicating positive sera testing being likely due to murine typhus alone.

Doxycycline is an effective antibiotic for both mild cases of leptospirosis and rickettsial infections; hence we suggest that in an area where the zoonotic infections co-circulate, it should be the first line antibiotic treatment in suspected cases of mild leptospirosis or rickettsiosis [13,26]. 

The limitation in our report is that we were unable to perform in vitro cultures and gene sequencing following negative PCR reactions, as recommended by previous studies [6,27]. The issues associated with cross-reactivity between species and persistence of antibodies after a previous infection, especially in areas where the infections co-exist, lead to problems with serological diagnosis and result in uncertainty of possible or true co-infections. Hence, a clear grading approach in diagnosis of rickettsial infections using combinations of culture and antigen, or DNA detection coupled with dynamic serology have proven to be useful [25]. Unfortunately, this is often unfeasible due to laborious cell culture techniques and potential requirement of biosafety level-3 laboratory containment (based on the risk assessment), which is costly. 

In conclusion, murine typhus should remain high on the list of differential diagnoses when faced with undifferentiated clinical symptoms and signs. Sharing a similar zoonotic risk, exposure to rats does not discriminate between murine typhus, leptospirosis, and scrub typhus, thus a need for a high index of clinical suspicion coupled with reliable confirmatory tests. Rapid tests are promising techniques, which may aid in early diagnosis, however, more studies are required to evaluate their reliability in endemic regions.
